# A novel component of the mitochondrial genome segregation machinery in trypanosomes

**DOI:** 10.15698/mic2016.08.519

**Published:** 2016-07-28

**Authors:** Anneliese Hoffmann, Martin Jakob, Torsten Ochsenreiter

**Affiliations:** 1 Institute of Cell Biology, University of Bern, 3012 Bern, Switzerland.; 2 Graduate School for Cellular and Biomedical Sciences, University of Bern, 3012 Bern, Switzerland.

**Keywords:** mitochondrial genome segregation machinery, TAC, kDNA, Trypanosoma brucei

## Abstract

We recently described a new component (TAC102) of the mitochondrial genome segregation machinery (mtGSM) in the protozoan parasite *Trypanosoma brucei*. *T. brucei* belongs to a group of organisms that contain a single mitochondrial organelle with a single mitochondrial genome (mt-genome) per cell. The mt-genome consists of 5000 minicircles (1 kb) and 25 maxicircles (23 kb) that are catenated into a large network. After replication of the network its segregation is driven by the separating basal bodies, which are homologous structures to the centrioles organizing the spindle apparatus in many eukaryotes. The structure connecting the basal body to the mt-genome was named the Tripartite Attachment Complex (TAC) owing its name to the distribution across three areas in the cell including the two mitochondrial membranes.

Proper segregation of the mt-genome during cell division is a general problem in biology and until now only a few components have been identified. In *T. brucei* four proteins (p166, p197, TAC40 and TAC102) have now been shown to be part of the TAC (Figure 1). Additionally, a monoclonal antibody (Mab22) has been described as a marker for the exclusion zone filaments, the cytoplasmic region of the TAC (Figure 1).

**Figure 1:**
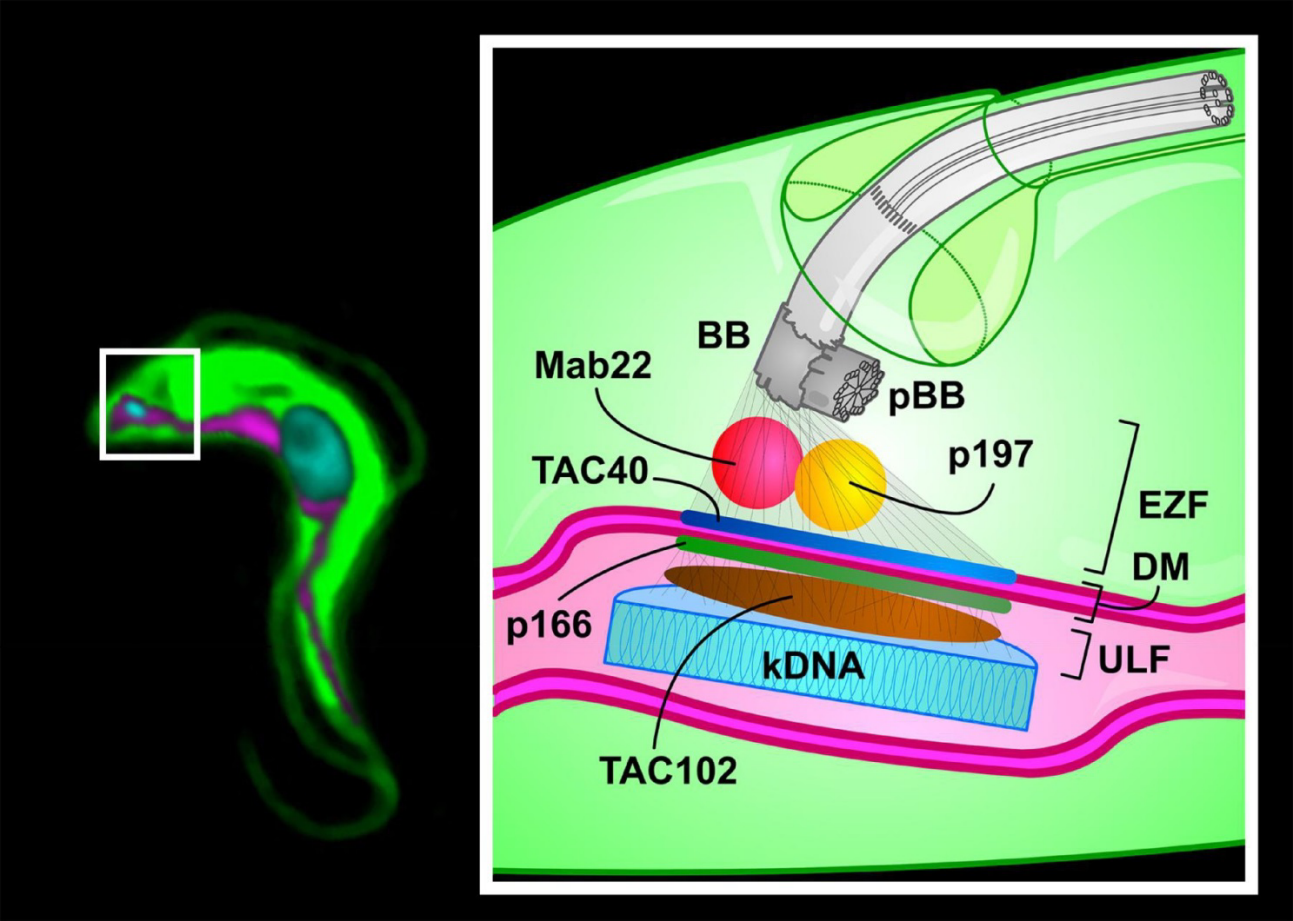
FIGURE 1: Model of the Tripartite Attachment Complex of a *T. brucei* cell. ** Left:** Montage of an epifluorescence image of a trypanosome cell. **Right:** The basal body (BB) is connected to the kDNA (**cyan**) via the Tripartite Attachment Complex (TAC). The TAC consists of the exclusion zone filaments (EZF), the differentiated membranes (DM) and the unilateral filaments (ULF). Four proteins (p197, TAC40, p166, TAC102) and one monoclonal antibody (Mab22) are shown as part of the TAC. The precise localization of p197 (**yellow**) and Mab22 (**red**) in the EZF is currently unknown. TAC40 (**blue**) is part of the outer mitochondrial membrane and p166 (**green**) localizes close to the inner mitochondrial membrane. TAC102 (**brown**) localizes to the ULF. Attached to the BB is the pro-basal body (pBB). **Green:** cytosolic part of the cell; **cyan**: DNA; **magenta**: mitochondrion.

Interestingly the TAC proteins are exclusively involved in mt-genome segregation without any additional functions. This is quite different from the multitude of functions that are currently associated with several of the mtGSM proteins in yeast or humans. Mdm10 in yeast, for example, which is involved in the assembly of the protein import machinery, is a part of the ERMES complex as well as crucial for proper mitochondrial morphology. Aside from TAC40, that has some similarities to Mdm10 in being a beta barrel protein of the outer mitochondrial membrane, there seems to be very little conservation in the components of the mtGSM. This includes TAC102 that does not show significant similarities to any protein outside the *Kinetoplastea*. This is rather surprising since many core components of other key organellar machineries like the protein import or oxidative phosphorylation complexes show considerable conservation throughout evolution, also in trypanosomes. The lack of conservation of the currently known TAC components including TAC102 might be explained by the difference in size and copy number and or the structure (linear vs. circular) of the mt-genomes. For the kinetoplastids the overall baroque organization and sheer size of the organellar genome (about 10^10^ kDa) might have led to a drastic adaptation of the mtGSM and thus to a loss of recognizable conservation. Nonetheless, it remains peculiar that such a central problem like mt-genome segregation should have evolved apparently many different molecular solutions.

TAC102 has a molecular weight of 102 kDa and is a basic protein (pI 9.4) containing a lysine rich region (653-756 aa) that is largely responsible for the basic isoelectric point. It is tempting to speculate that the lysine rich region might constitute a DNA binding domain. However, so far we have been unable to provide evidence that the recombinant TAC102 protein indeed shows any DNA binding affinity. Furthermore, this domain is only present in trypanosomes and absent in the other *Kinetoplastea* like *Crithidia* or *Leishmania* where the TAC102 homologues have a predicted acidic pI of 4.6. Taken together we do not think that the lysine rich region has an essential function in kDNA segregation, which can easily be tested by deletion mutants in the future.

A surprising observation we made was the apparent contradiction in solubility of the TAC102 protein by different detergents. Using low concentration of digitonin we could show that TAC102 solubilizes from the mitochondrial matrix similar to a known marker protein. In a different experiment TAC102 strongly associated to the TAC in detergent isolated flagella (0.5% Triton-X100) a typical feature of TAC proteins. We speculate that some portion of TAC102 is strongly bound to the TAC and might become insoluble once the complex has been isolated, while another portion is readily soluble. Alternatively, the harsh cytoskeletal extraction might lead to an insoluble TAC, which would also explain why all other known components also remain in the TAC under these conditions. Interestingly, while the kDNA requires TAC102 for proper segregation, TAC102 does not require the kDNA to remain associated with the TAC, which can easily be seen in isolated flagella treated with DNAseI.

When we analyzed the different deletion mutants of TAC120 we could show that the C‐terminus is required for proper localization to the organelle and the TAC, while the N‐terminus is dispensable for this function. However, tinkering with the TAC102 N‐terminus lead to a phenotype where small kinetoplasts could be seen at non-conventional positions throughout the mitochondrion, co‐localizing with extra TAC102 foci. The observation of extra kDNA particles is not unusual but it’s the first time that a mutant protein can be directly attributed to and co-localized with the appearance of the extra structures. We can speculate that the N-terminal mutant TAC102 is still able to initiate the assembly of a TAC subcomplex in the mitochondrial matrix that allows kinetoplast formation. However, due to the compromised N-terminus of the protein, the complex is unable to connect to the upstream components of the TAC and thus is “free floating” in the mitochondrion.

One of the questions for future research is how the large, two membrane spanning TAC is assembled during the cell cycle. In first experiments we have now shown that TAC102 quickly disappears at the new basal body kDNA connection during RNAi knockdown experiments, while it remains stably associated with the old basal body indicating that turnover at the existing structure is very low. If this observation holds for the other components it would argue for a *de novo* assembly of the TAC during cell cycle. It will be interesting to see if the assembly is organized in some kind of hierarchy and how this might be regulated. Phosphorylation sites on TAC102 and on other TAC components (p197) already hint towards a possible regulation mechanism. Along these lines, it is worth investigating the role of the basal body and/or pro-basal body in the assembly of the TAC. Finally, although a few components of the TAC have been identified and we do not know how many more will be discovered, we can predict that some functions are still missing including the connection between the inner and outer mitochondrial membrane and the mitochondrial kinetochore structure linking the kDNA to the TAC.

